# LASP1, a Newly Identified Melanocytic Protein with a Possible Role in Melanin Release, but Not in Melanoma Progression

**DOI:** 10.1371/journal.pone.0129219

**Published:** 2015-06-10

**Authors:** Anjana Vaman V. S., Heiko Poppe, Roland Houben, Thomas G. P. Grunewald, Matthias Goebeler, Elke Butt

**Affiliations:** 1 Institute of Clinical Biochemistry and Pathobiochemistry, University Hospital Würzburg, Würzburg, Germany; 2 Department of Dermatology, University Hospital Würzburg, Würzburg, Germany; 3 Laboratory for Pediatric Sarcoma Biology, Institute of Pathology, Ludwig Maximilians University Munich, Munich, Germany; Rutgers University, UNITED STATES

## Abstract

The LIM and SH3 protein 1 (LASP1) is a focal adhesion protein. Its expression is increased in many malignant tumors. However, little is known about the physiological role of the protein. In the present study, we investigated the expression and function of LASP1 in normal skin, melanocytic nevi and malignant melanoma. In normal skin, a distinct LASP1 expression is visible only in the basal epidermal layer while in nevi LASP1 protein is detected in all melanocytes. Melanoma exhibit no increase in LASP1 mRNA compared to normal skin. In melanocytes, the protein is bound to dynamin and mainly localized at late melanosomes along the edges and at the tips of the cell. Knockdown of LASP1 results in increased melanin concentration in the cells. Collectively, we identified LASP1 as a hitherto unknown protein in melanocytes and as novel partner of dynamin in the physiological process of membrane constriction and melanosome vesicle release.

## Introduction

Melanocytes are specialized cells of neuroectodermal origin that produce melanosomes, i.e. vesicles in which melanin pigment is synthesized to protect the DNA of epidermal cells against UV light-induced damage. Melanosomes are mainly produced around the nucleus of melanocytes, then transported along microtubules and actin filaments to the dendrite tips of the cells and subsequently shed from the plasma membrane to finally become internalized by keratinocytes [1, 2]. The mechanisms involved in melanosome maturation, transport and release are highly dependent on molecular motors along with multi-protein assemblies and cytoskeletal rearrangements [3].

We previously identified the LIM and SH3 protein 1 (LASP1) to be strongly expressed in epidermal basal cells [4]. LASP1 is a scaffolding protein involved in cell migration and proliferation and preferentially localized at focal contacts and along the membrane edges of the cell [5]. LASP1 harbours an N-terminal LIM domain, followed by two actin-binding nebulin repeats, a linker domain and a C-terminal Src homology 3 (SH3) domain [6]. Various cytoskeleton proteins have been identified that bind to LASP1: F-actin [7], kelch-related protein [8], zyxin [9], lipoma preferred partner [7], zona occludens protein 2 (ZO2) [10], dynamin [10], VASP [7], CRKL [11] and CXCR2 [12], respectively.

Phosphorylation by protein kinases A and G at serine 146 reduces binding of LASP1 to F-actin, increases cytosolic distribution and enables nuclear shuttling by binding to zona occludens protein 2 (ZO2) [10]. Phosphorylation at tyrosine 171 by Abl-kinase blocks LASP1 translocation to focal complexes [13].

LASP1 is overexpressed in a multitude of cancers. Several studies demonstrated that LASP1 expression and nuclear localization positively correlated with malignancy, tumor grade, and metastatic lymph node status (for review see: [14]).

A previously reported physiological LASP1-depending process that resembles melanosome release by melanocytes is the secretory HCl response in gastric parietal cells [15, 16]. Stimulation of acid secretion involves the translocation of H^+^/K^+^-ATPase vesicles from the cell cortex to the apical membrane of the parietal cell, followed by vesicle fusion with the plasma membrane.

We therefore studied LASP1 expression in skin tissue samples, in normal human epidermal melanocytes (NHEMs) and in melanoma cell lines and identified LASP1 as a yet unknown protein in melanocytes and as novel partner of dynamin in the complex process of melanosome vesicle release at the dendrite tips.

## Materials and Methods

### Tissue samples

112 archived formalin-fixed and paraffin-embedded (FFPE) tissue specimens (including 58 primary malignant melanomas, 20 independent melanoma metastases, 29 melanocytic nevi and 5 normal skin samples) were obtained by surgical excision for either therapeutic or diagnostic purposes and had undergone routine histology at the Department of Dermatology, University Hospital Würzburg, Würzburg, Germany. The institutional review board of the University Hospital Würzburg, Germany, specifically approved this study and waived the need for written informed consent from the donors.

### Immunohistochemistry

Immunohistochemistry of tissue samples was carried out on 4 consecutive sections as described earlier [17]. Tissue sections were incubated overnight at 4°C with antibodies recognizing LASP1 (1:1000) [7] and MART1 (Dako, M7196, 1:200) diluted in PBS.

Immunostained sections were evaluated by two independent scientists to define the percentage of LASP1-positive cells and to determine nuclear and cytosolic immunoreactivity. Cytosolic LASP1 expression was quantified in analogy to the scoring of the hormone receptor Immune Reactive Score (IRS), ranging from 0–12 according to Remmele et al. and as described in detail for LASP1 in breast cancer [4, 18]. For statistical discrimination, samples scored with cytosolic LASP1-IRS <3 were classified as LASP1-negative and those with LASP1-IRS ≥3 as LASP1-positive. Nuclear LASP1 positivity was scored by determining the percentage of positive nuclei regardless of cytosolic LASP1 immunoreactivity. Samples were considered to be nuclear-positive if 10% or more cells showed nuclear LASP1 staining.

### Cell lines and culture conditions

Human primary melanoma cell lines LOXIMVI, M14, MDAMB435, SKMel5, SKMel2, M19-Mel [19], UACC62, UACC257 [20] and the subcutaneous metastasis cell line MaMel2 [21] were cultured in DMEM supplemented with 10% fetal calf serum and penicillin/streptomycin (100 U ml^−1^, Gibco, Darmstadt, Germany). Normal human epidermal melanocytes (NHEMs) were purchased from PromoCell (Heidelberg, Germany) and cultured in HAM's F10 medium supplemented with 20% fetal bovine serum, glutamine, ITS premix, 12-O-tetradecanoylphorbol-13-acetate, IBMX, and cholera toxin (Gibco).

### Western blot (WB)

For Western blotting, cells were lysed in 2x Laemmli sample buffer (65.8 mM Tris-HCl, pH 6.8, 26.3% (w/v) glycerol, 2.1% SDS, 0.01% bromophenol blue). Equal amounts of protein, according to cell count, were resolved by 10% SDS-PAGE. After blotting on a nitrocellulose membrane (Schleicher und Schuell, Kassel, Germany) and blocking with 3% nonfat dry milk (Biorad, Munich, Germany) in 10 mM Tris, pH 7.5, 100 mM NaCl, 0.1% (w/v) Tween 20, the membrane was first incubated with a self-generated polyclonal rabbit antibody against LASP1 (diluted 1:1000) [4], a mouse monoclonal antibody against dynamin (diluted 1:200; Santa Cruz, Heidelberg, Germany), or rabbit polyclonal antibodies against TRP1 (diluted 1:200; Santa Cruz), tyrosinase (diluted 1:200, Santa Cruz) or β-actin (diluted 1:5000, Sigma-Aldrich, Deisenhofen, Germany). Thereafter, membranes were incubated with horseradish peroxidase-coupled goat anti-rabbit or anti-mouse IgG (diluted 1:5000; Biorad, Munich, Germany). Signals were subsequently visualized by ECL (Amersham Biosciences, Freiburg, Germany) and quantified by densitometry using the ImageJ software (NIH, Bethesda, USA).

### p53 status

Most of the melanoma cell lines used in this study belong to the well- characterized NCI-60 panel for which p53 mutation data are available http://p53.free.fr/Database/Cancer_cell_lines/NCI_60_cell_lines.html. The remaining cell lines were analyzed by sequencing the p53 exons 5–8. Genomic DNA was extracted from the different melanoma cell lines and subjected to semi-nested PCR using previously reported primers [22]. The amplicons were analyzed by Sanger sequencing.

### Microarray analysis

Publicly available gene expression data of n = 604 individual tissue samples were retrieved from the Gene Expression Omnibus (GEO). All data were generated on Affymetrix HG-U133plus2.0 microarrays. Accession codes: n = 353 normal body atlas GSE3526 [23]; n = 166 melanoma GSE10282, GSE15605, and GSE35640; n = 85 normal skin GSE14905 and GSE13355. Expression data were manually revised for their correct annotations and simultaneously normalized by Robust Multi-array Average (RMA) [24] using custom brainarray (v18 ENTREZG) CDF files yielding one optimized probe-set for each gene corresponding to the ENTREZ gene ID as described elsewhere [25–27]. Statistical analyses were performed using Prism v5.0b (GraphPad Software, Inc.). *P* values < 0.05 were considered statistically significant (two-tailed student’s *t*-test with Welch’s correction).

### Immunofluorescence

For immunofluorescence microscopy cells were grown until 70% confluence on glass coverslips. Cells were fixed in 4% (w/v) paraformaldehyde in PBS, permeabilized with 0.1% (w/v) Triton X-100 in PBS, and then stained with affinity-purified rabbit polyclonal antibody against LASP1 (diluted 1 μg/ml) [4] in combination with either mouse monoclonal antibodies against dynamin (diluted 1:20, Santa Cruz) or tyrosinase (diluted 1:20, Santa Cruz), followed by secondary Cy3-labeled anti-rabbit antibody and Cy5-labeled anti-mouse antibody (diluted 1:250, Dianova, Hamburg, Germany). Nuclei were visualized with DAPI (diluted 1:1000, Sigma-Aldrich). Omnifocal analyses were done with a Biorevo BZ-9000 (Keyence, Neu-Isenburg, Germany). The obtained data were processed by using Photoshop (Adobe Systems, San Jose, CA, USA).

### Preparation of nuclear and cytosolic cell fractions

For preparation of cell fractions, cells were harvested and washed twice in PBS. Isolation of nuclei and cytosol was carried out using NE-PER nuclear and cytoplasmic extraction reagents (Pierce, Bonn, Germany) following the manufacturer's instructions.

### siRNA transfection

Transfection for LASP1 knockdown was performed using metafectene (Biontex Laboratories GmbH, Martinsried, Germany) according to the manufacturer’s protocol for suspension culture. Briefly, suspended cells were used at a density of 1x10^5^ cells/ml and incubated directly with the pre-incubated mixture of 12 μl metafectene and 1.2 μM control siRNA (AllStar negative control, Qiagen, Hilden, Germany) or LASP1 siRNA (5´-AAG CAT GCT TCC ATT GCG AGA -3`(bases 80–100). Cells were cultured at 37°C under 5% CO_2_ atmosphere for 72h.

### Proliferation assay

Cell viability was determined by the CellTiter-Glo Luminescent Cell Viability Assay (Promega, Mannheim, Germany) according to the manufacturer´s instructions. In brief, cells transfected with either siRNA against LASP1 or nonsense siRNA were cultured for 72h. Cell proliferation was expressed as percentage of control cells. Four independent experiments were performed, each with 8 replicates. In addition, cells were counted with a Neubauer chamber. Successful depletion of LASP1 was confirmed by Western blot.

### Adhesion assay

Cells were seeded in 48-well plates (4x10^4^ cells per well) and allowed to attach for 4h at 37°C (50% adhesion for control cells). Non-adherent cells were removed by gentle washing with PBS and wells were refilled with 100 μl medium. Cells were counted using the CellTiter-Glo Luminescent Cell Viability Assay (Promega) according to the manufacturer´s instruction. Wells with non-washed-off cells served as 100% control. Six independent experiments, each with 5 replicates, were performed. Successful knockdown of LASP1 was confirmed by Western blot.

### Migration assay

Cellular migration was assessed by a modified Boyden chamber assay (transwell 8 μM pore size, Corning Star, Cambridge, MA, USA). Cells were serum-starved overnight, trypsinized, adjusted for viability, counted, and re-suspended in serum-free medium at a density of 1.5x10^6^ cells/ml. Prior to the experiment, the lower surface of the filter membrane was coated with 100 μl fibronectin solution (5 μg/ml; Sigma-Aldrich) for 15 min as a chemo-attractant while the inner filter chambers were coated with 100 μl 10% FCS in DMEM medium for 30 min. 500 μl DMEM medium with 10% FCS was added to the lower chamber of each transwell. 100 μl cell suspensions (1.5x10^5^ cells) were placed into the upper filter chambers. Cells were incubated for 6h (37°C; 5% CO_2_) to allow migration. Non-migrated cells from the upper chamber were removed using a cotton swab. Migrated cells at the lower surface of the membranes were stained in 200 μl 1% (w/v) crystal violet in 2% ethanol for 30 sec and rinsed twice afterwards in distilled water. Cell-associated crystal violet was extracted by incubating the membrane in 200 μl 10% acetic acid for 20 min and measured at 595 nm absorbance using a plate reader (Molecular Devices, Crawley, UK). Three independent experiments, each with 6 replicates, were performed.

### Melanin assay

Cells were transfected and seeded at a density of 1x10^5^ cells/ml in 6-well plates. 72h post transfection, cells were harvested, washed in PBS and lysed in 1N NaOH in 10% DMSO. Cellular melanin was dissolved by incubating the homogenate at 80°C for 2h and then centrifuged at 500x*g* for 5 min. Absorbance of the supernatant was measured at 405nm. Synthetic melanin (Sigma) was used as standard, ranging from 0–80 μg/ml.

### Melanosome isolation by sucrose density gradient

Melanosomes were purified by ultracentrifugation as described [28]. For our experiments we reduced all quantities to 1/10 of the original protocol. Briefly, the cellular homogenate was layered on a discontinuous gradient of 1.0, 1.2, 1.4, 1.5, 1.6, 1.8, and 2.0 M sucrose (in 10 mM Hepes, pH 7.0) and centrifuged at 100,000x*g* in a Beckman SW 55 Ti swinging-bucket rotor for 1h at 4°C. Melanosomes that localized at various layers of the gradient were recovered by pipette and analyzed by Western blot.

### Pulldown

MaMel2 cells (1x10^6^ cells) were lysed in 500 μl hypotonic buffer (Complete Mini) for 30 min at 4°C, followed by mechanic lysis with a 29 1/2 gauge needle. After centrifugation for 30 min at 20,000x*g*, the supernatant was incubated for 2h with GST-LASP1 beads or control GST-beads, washed three times with PBS and analysed by SDS-PAGE and Western Blot for bound proteins.

### Statistics

Comparisons of patient characteristics between LASP1_*positive*_ and LASP1_*negative*_ protein expression were performed using Chi-Square test. Student’s t test was applied to determine effects of siRNA knockdown on cell proliferation, adhesion and migration. A result was considered significant at p≤0.05.

## Results

### LASP1 expression in normal skin and in nevi

We assessed LASP1 protein expression patterns by immunohistochemistry in normal skin and melanocytic nevi with antibodies against LASP1 and MART1 in consecutive tissue sections.

**Fig 1:**
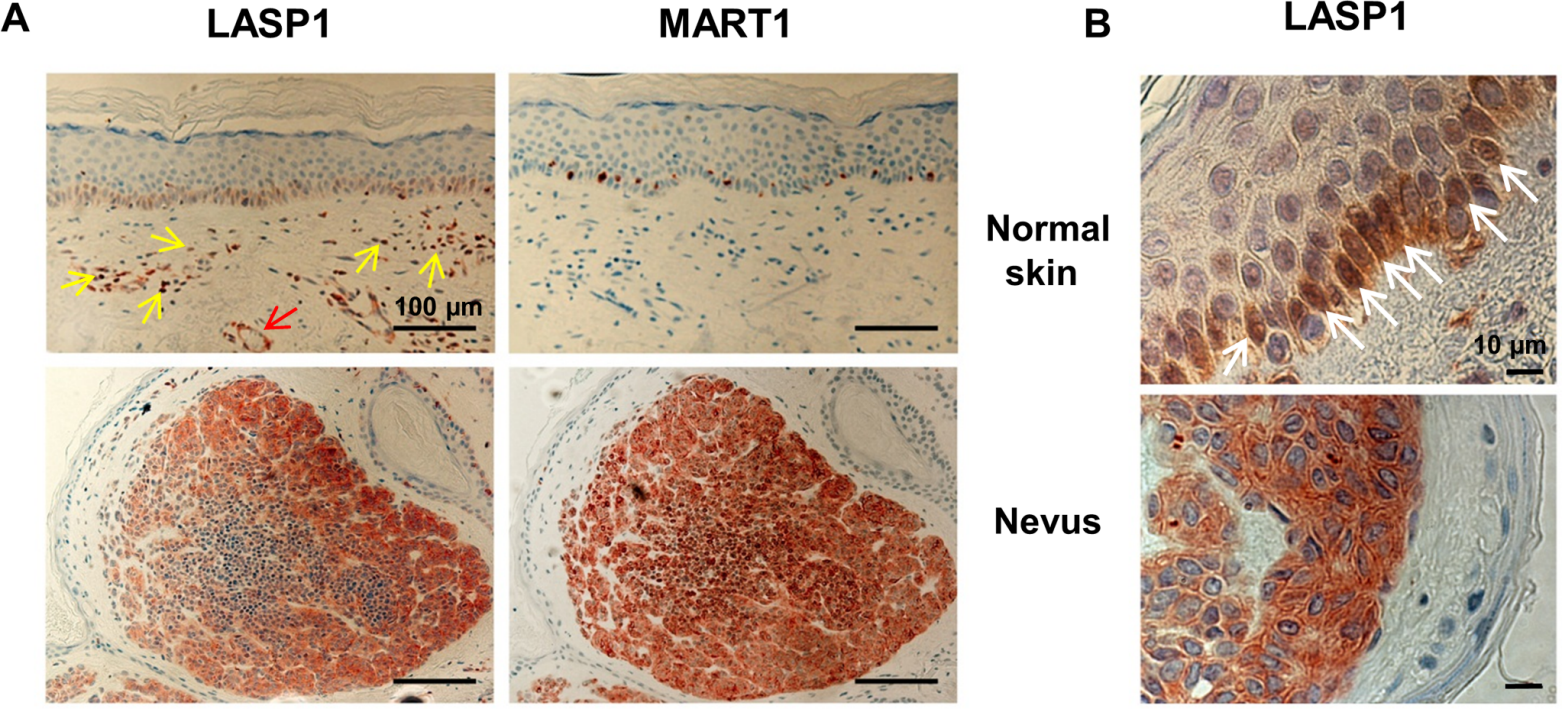
LASP1 expression in normal skin and nevi. (A) Representative immunohistochemical staining of consecutive sections from normal skin and from a representative melanocytic nevus for LASP1 and the melanocytic antigen MART1 (magnification 20x; Scale bar = 100 μm). Stratum basale of normal skin and nevus shows distinct LASP1-positivity. Histiocytes (yellow arrows) and a transverse cut blood vessel (red arrow) in the dermis display LASP1 expression. (B) Immunohistochemical LASP1 staining of normal skin and nevus at higher magnification (100x; Scale bar = 10 μm). Nuclear LASP1-positive cells of the epidermal basal layer in normal skin are marked with arrows. Hematoxylin is used for counterstaining.

LASP1 shows a positive immunostaining in the epidermal basal layer of normal skin (stratum basale), composed of basal keratinocytes and pigment producing melanocytes (Fig 1A upper left). The presence of interspersed melanocytes is confirmed by melanocyte-specific MART1 staining in the consecutive section (Fig 1A upper right). Basal cells divide, differentiate and migrate towards the surface. In these cells, the nuclei are very frequently LASP1-positive (upper Fig 1B). Unlike to the stratum basale, the differentiating keratinocytes of the stratum spinosum and the stratum granulosum are LASP1 negative (Fig 1A upper left). In the dermis, histiocytes (yellow arrows) and blood vessels with vascular smooth muscle cells (red arrow) also display LASP1-positivity (Fig 1A upper left). This has been observed earlier [29, 30].

Benign melanocytic nevi show high LASP1 protein expression in MART1-positive nevus cells without nuclear LASP1 staining (lower Fig 1A and 1B).

### LASP1 expression level in NHEM and melanoma cell lines

**Fig 2:**
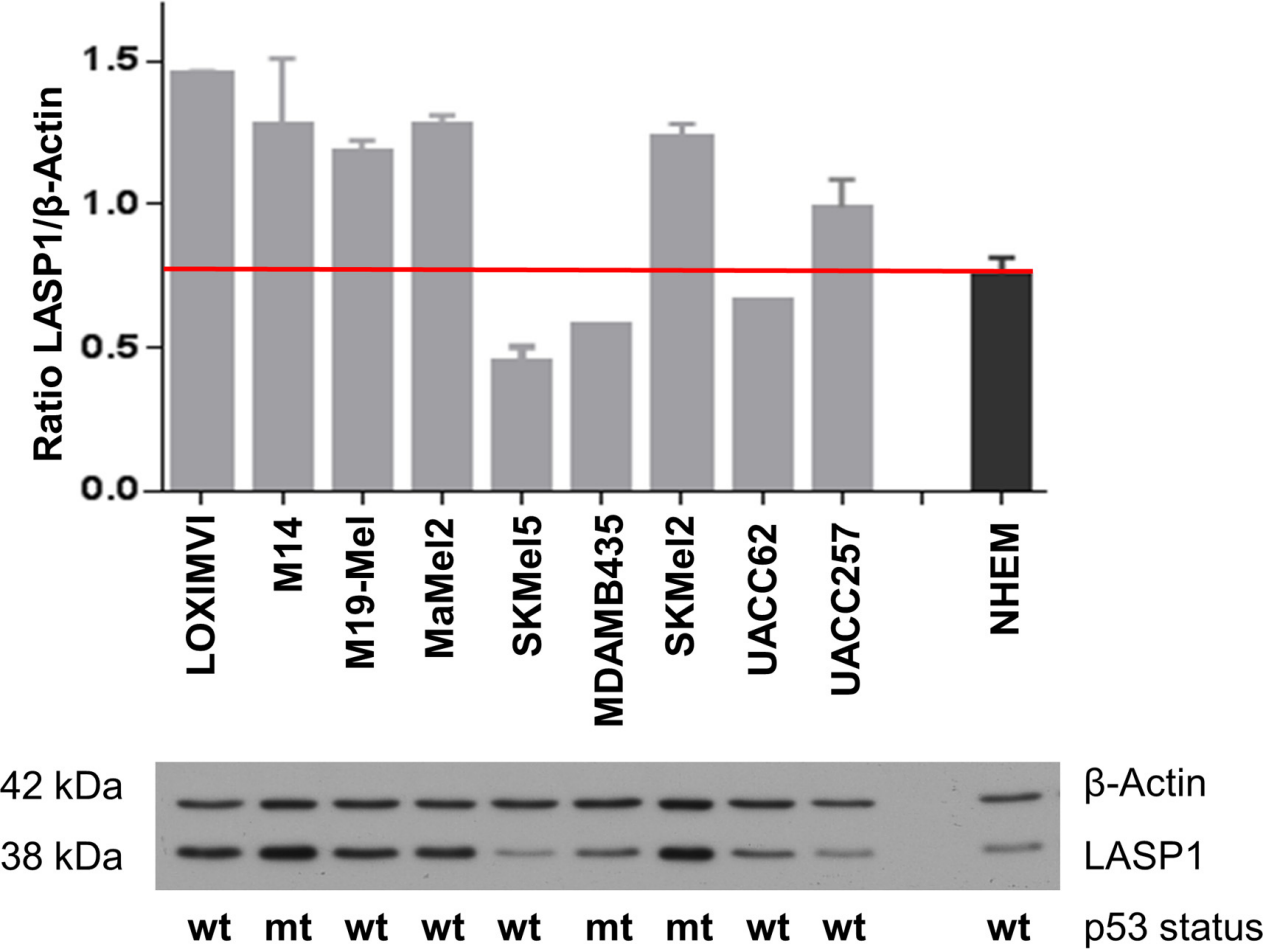
LASP1 expression in melanoma cell lines. Expression of LASP1 and β-actin in melanoma cell lines and in normal human epidermal melanocytes (NHEMs) was determined by Western blotting (10 μg protein per lane). Bar graphs represent mean and SEM integrated optical density of Western blot bands from three individual experiments. The mutational status of the p53 oncogene is indicated for each cell line (wt wildtype; mt, mutant).

Next, we used Western blot (WB) to analyze the expression of LASP1 in cultured normal human epidermal melanocytes (NHEMs) and different melanoma cell lines, which revealed LASP1 expression in NHEMs as well as in all studied melanoma cell lines (Fig 2). For further experiments, we chose MaMel2, a melanin-producing cell line derived from a subcutaneous melanoma metastasis that showed higher LASP1 levels than NHEMs, and UACC257, a slightly pigmented primary melanoma cell line with a LASP1 expression level similar to NHEMs.

Since LASP1 transcription is regulated in part by the tumor suppressor p53 [31] we validated and included the p53 status of the analyzed cell lines. However, no apparent correlation between LASP1 expression levels and p53 mutations was observed (Fig 2).

### Knockdown of LASP1 is influencing migration and proliferation

**Fig 3:**
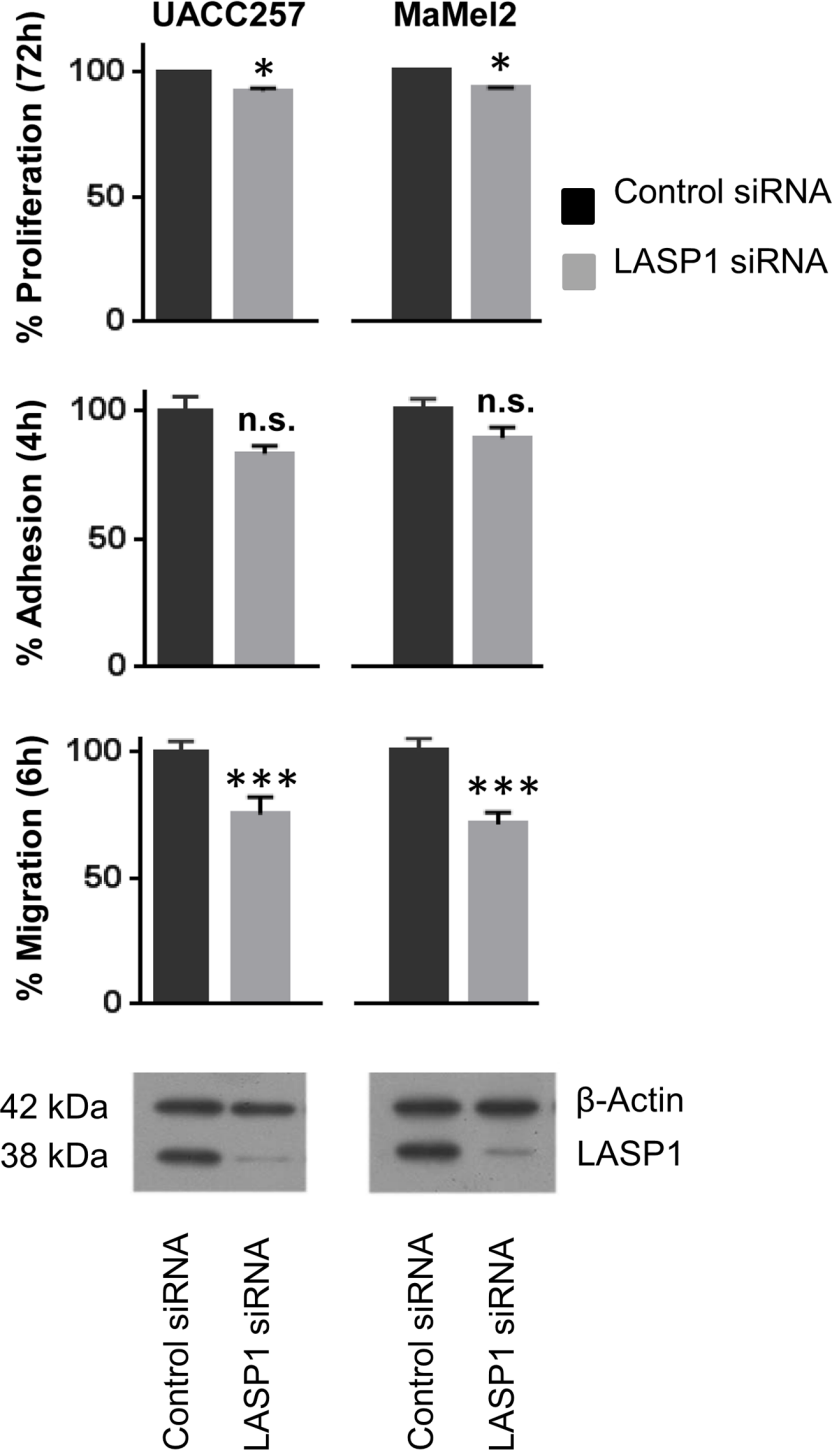
LASP1 knockdown affects cell migration and proliferation. For analysis of proliferation, melanoma cell lines transfected with LASP1 siRNA and control siRNA were seeded in 48-well-plates and incubated for 72h. Cells were counted with MTT viability assay and by Neubauer chamber. Bars, SEM; * Student´s *t* test, p<0.05; *versus* control. For investigation of adhesion properties, LASP1-depleted cells were seeded in 48-well-plates and incubated for 4h. Non-adherent cells were washed-off, adherent cells were counted with Promega Glo. Bars, SEM; n.s. p≥0.05, versus control. For migration analysis, LASP1-depleted cells were seeded in modified Boyden chambers and incubated for 6h. Migrating cells were fixed with paraformaldehyde and stained with crystal violet. The absorbance was measured. Bars, SEM; *** Student´s *t* test, p≤0.001; *versus* control. A representative Western blot for LASP1 knockdown (~75%) in both cell lines is shown.

To functionally assess the role of LASP1 in melanocytes and melanoma, we used NHEMs and two melanoma cell lines, i.e. UACC257 and MaMel2. Cell lines were transfected with siRNA against LASP1 or control siRNA. A profound reduction of ~75% of LASP1 protein expression with maximum silencing after 72h is observed in the melanoma cells (Fig 3). Attempts to transfect NHEM cells, either by transfection reagents (Metafectene, HiPerfect) or by Amaxa nucleofector electroporation failed.

Since LASP1 has been shown to have a functional role in cell viability, motility and tumor dissemination [13, 32–34], we also investigated cell migration, adhesion and proliferation in melanoma cells before and after LASP1 silencing by using a modified Boyden chamber assay, a fibronectin adhesion assay and an MTT viability test, respectively. Overall, only very minor consequences could be measured. While adhesion was not affected by LASP1 knockdown in MaMel2 and UACC257 cells (*p*>0.05), we observed a distinct reduction of the migratory potential (~25%, *p*≤0.005) and a modest, but significant inhibition of proliferation (~10%, *p*≤0.001) by MTT assay and by cell counting (Fig 3).

### LASP1 binds to dynamin at the dendrite tips and contributes to melanin release

**Fig 4:**
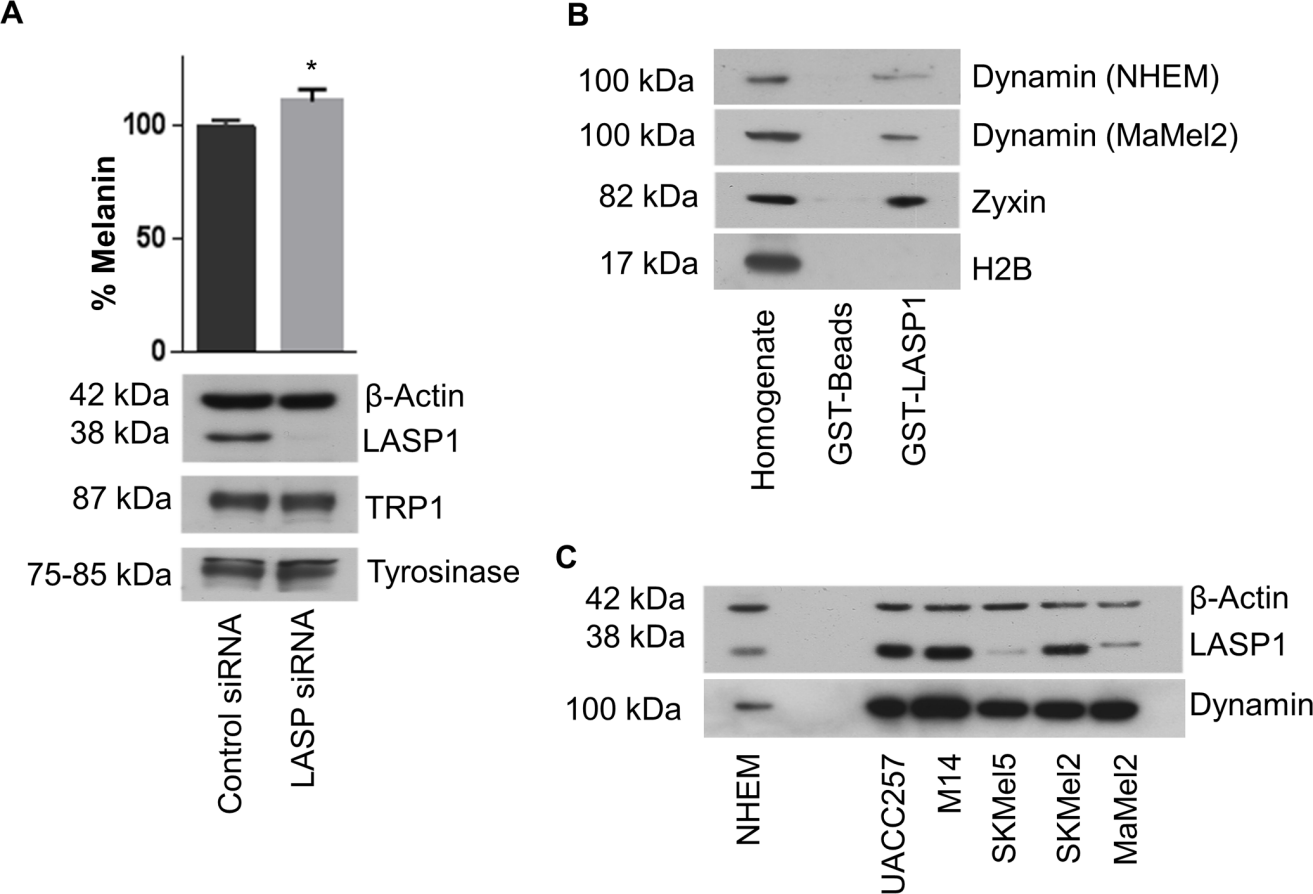
Dynamin is a LASP1 binding partner. (A) Melanin concentration before and after LASP1 knockdown in MaMel2 cells. LASP1 knockdown efficiency was controlled by Western blot. Expression levels of tyrosinase and TRP1 are not affected. (B) Western blot analysis of dynamin, zyxin (positve control) and histone H2B (negative control), pulled-down with GST control beads and GST-LASP1 from MaMel2 and NHEM homogenate. (C) Western blot analysis of dynamin and LASP1 expression in melanoma cell lines and normal human epidermal melanocytes (NHEM). β-Actin served as loading control.

As shown in Fig 1 LASP1 is highly expressed in melanocytic nevi, which are benign proliferations of melanin-producing melanocytes. Due to its domain structure, LASP1 can bind to several proteins involved in melanosome vesicle trafficking, e.g. F-actin [35, 36] and dynamin [10, 37]. We speculated whether LASP1 might have a specific function in melanocytes and therefore examined the melanin content of pigmented MaMel2 cells before and after LASP1 knockdown. As shown in Fig 4A, LASP1 silencing induced a moderate, but significant elevation of the melanin concentration in these cells (~10%, *p* = 0.03). Expression levels of tyrosinase and TRP1, two major players in melanogenesis, were not affected by LASP1 knockdown.

Next we used immunofluorescence to examine a possible co-localization of LASP1 with dynamin, a putative protein identified in melanosomes [37] and well-known for its involvement in clathrin-coated vesicle trafficking [38] as well as with tyrosinase, a stage III and IV melanosome marker.

**Fig 5:**
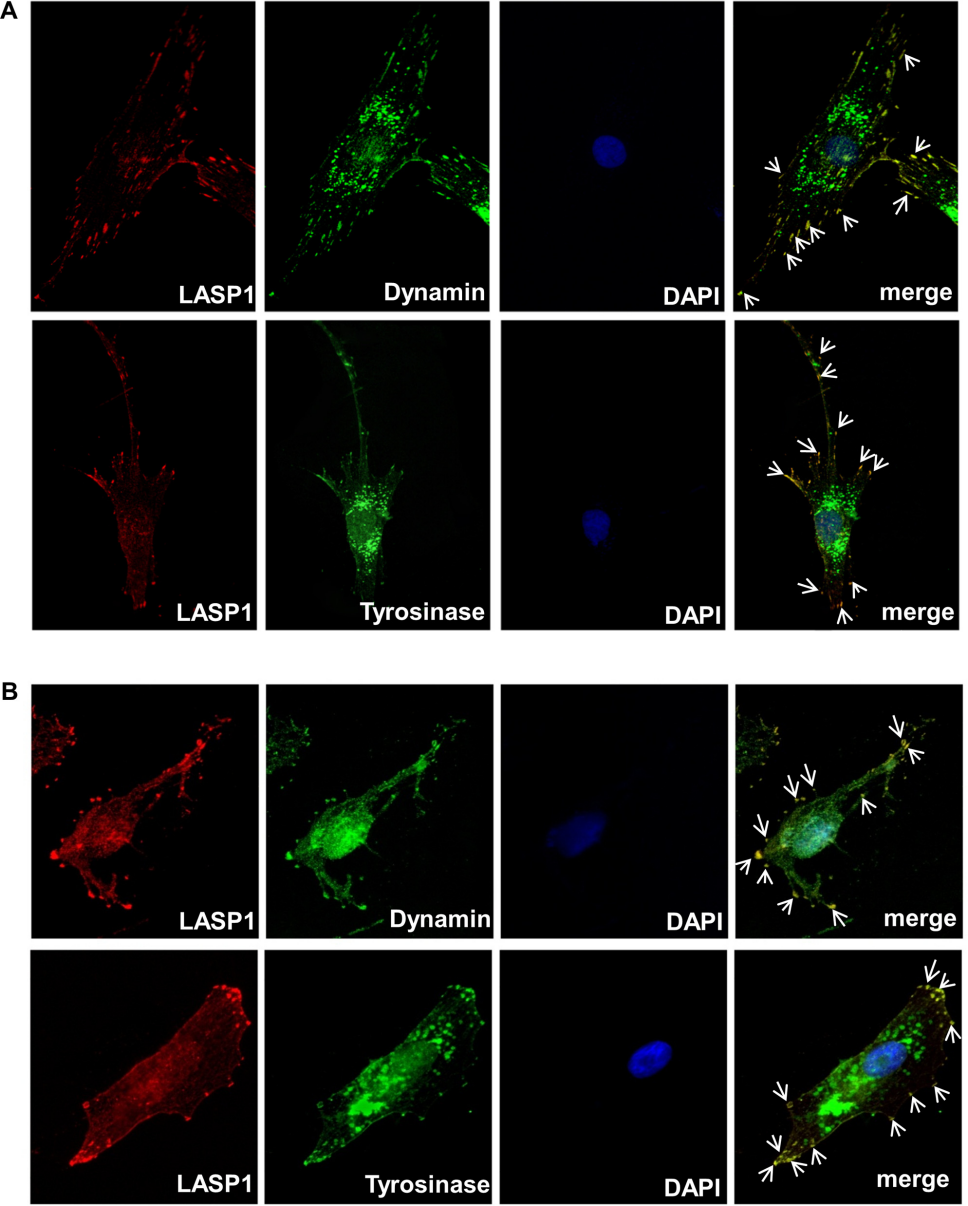
Immunofluorescence of LASP1, dynamin and tyrosinase in melanoma cells. NHEM (A) and MaMel2 (B) cells were fixed, permeabilized and stained for LASP1 (red), dynamin or tyrosinase (both green) and nuclei (DAPI, blue). In merged cells, yellow visualizes a co-localisation of LASP1 with dynamin or tyrosinase at the tips of the cell (indicated by white arrows).

Immunofluorescence imaging with NHEM (Fig 5A) and MaMel2 cells (Fig 5B) verified the presence of dynamin and tyrosinase mainly at the Golgi apparatus around the nucleus and along the cell membrane and tips of the cell. In NHEM cells, a clear punctual LASP1, dynamin and tyrosinase staining corresponding to few and discrete melanosomes is observed while in highly pigmented MaMel2 cells increased melanocyte production is represented by a more diffuse and bright staining of the cytosol mainly for dynamin and tyrosinase. Co-localization with LASP1 is only observed at the cell membrane but not at the Golgi complex, suggesting a possible involvement of LASP1 in melanosome release. Control experiments without primary antibodies revealed no staining with the secondary antibodies.

Next we asked whether the observed co-localization of LASP1 with dynamin is due to direct or indirect physical interaction. To this end, we performed pull-down assays demonstrating definite binding of dynamin to GST-LASP1 beads in MaMel2 and NHEM homogenates (Fig 4B). Binding to zyxin, a well-known LASP1 interacting partner [9], served as positive control while no binding was observed with histone H2B (negative control). Similar results were obtained with UACC257 cells (data not shown). Interestingly, all tested melanoma cell lines expressed dynamin to a higher extent than NHEM cells, independently of their pigmentation status (Fig 4C).

### Identification of LASP1 and dynamin in melanosomes

**Fig 6:**
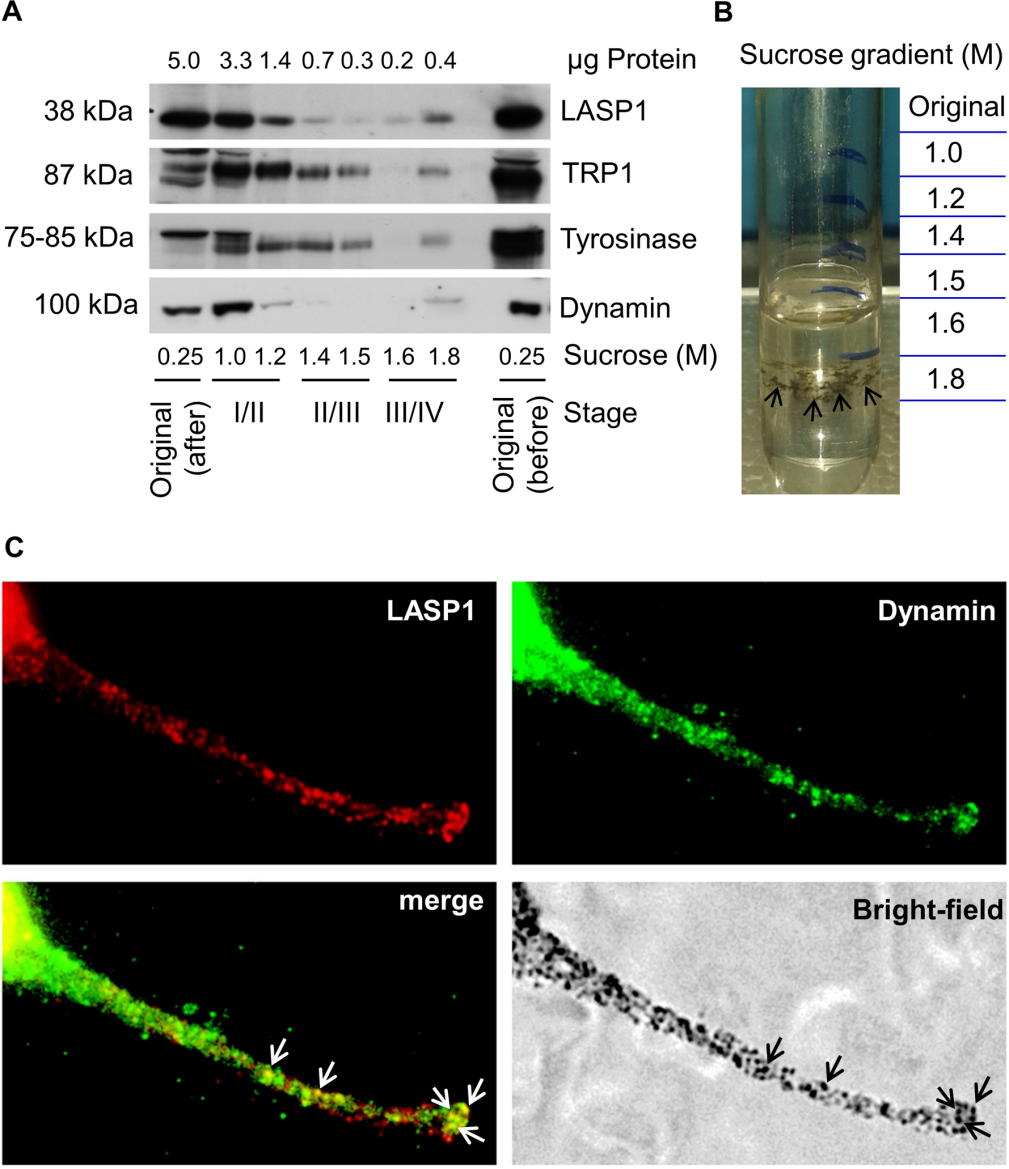
Co-localization of LASP1 and dynamin in melanosomes. (A) MaMel2 cells were prepared as described in “Materials and Methods” and loaded on a sucrose density-gradient. Equal fraction volumes were subjected to SDS/PAGE and immunoblotted for LASP1, TRP1, tyrosinase and dynamin. “Original before” indicates the loading sample in 0.25 M sucrose before centrifugation. “Original after” indicates the loading sample fraction after centrifugation. The protein concentrations of the fractions and the corresponding melanosome stages are stated. (B) Sucrose density gradient tube with concentrated melanosomes (indicated by black arrows) in the 1.8 M sucrose gradient fraction. (C) LASP1 (red) and dynamin (green) immunofluorescence at the dendrite tip of a MaMel2 cell. Co-localization of the merged LASP1-dynamin complex (yellow) with melanosomes (black arrows) in the bright-field image at the dendrite tip of the MaMel2 cell.

Melanosomes mature within the melanocyte through four morphologically distinct stages [39] that can be separated by sucrose density-gradient ultracentrifugation [28]. While 1.0 M and 1.2 M sucrose fractions contain the majority of stage I and stage II melanosomes, stages III and IV are mainly detected in the 1.6 M and 1.8 M fractions. Immunoblot analysis of the fractions demonstrated the highest protein concentration in the low density 1.0 M and 1.2 M sucrose fractions (Fig 6A). High dynamin levels in this range reflect involvement of the protein in melanosome budding from the Golgi complex. Tyrosinase was present in almost all parts of the gradient while—in agreement with earlier publications [40]—TRP1 was more abundant in fractions containing early-stage melanosomes (Fig 6A). The high LASP1 concentration in the low density sucrose fractions does not represent LASP1 localization in early melanosomes but reflects incomplete protein solubilization of this cytoskeleton-associated protein in the detergent-free melanosome preparation buffer, as suggested by still high LASP1 levels in the “original” after centrifugation (Fig 6A). The data is further supported by our immunofluorescence images, showing no LASP1 co-localization with tyrosinase and dynamin along the Golgi apparatus around the nucleus (Fig 5).

In contrast, however, LASP1, following a gradual decrease along the gradient, increased again in the 1.8 M sucrose fraction, indicating discrete presence in stage IV melanosomes (Fig 6A). Dynamin, tyrosinase and TRP1 also localized to this fraction and in two out of four experiments, even dark melanosomes were visible in the 1.8 M sucrose fraction (Fig 6B).

**Fig 7:**
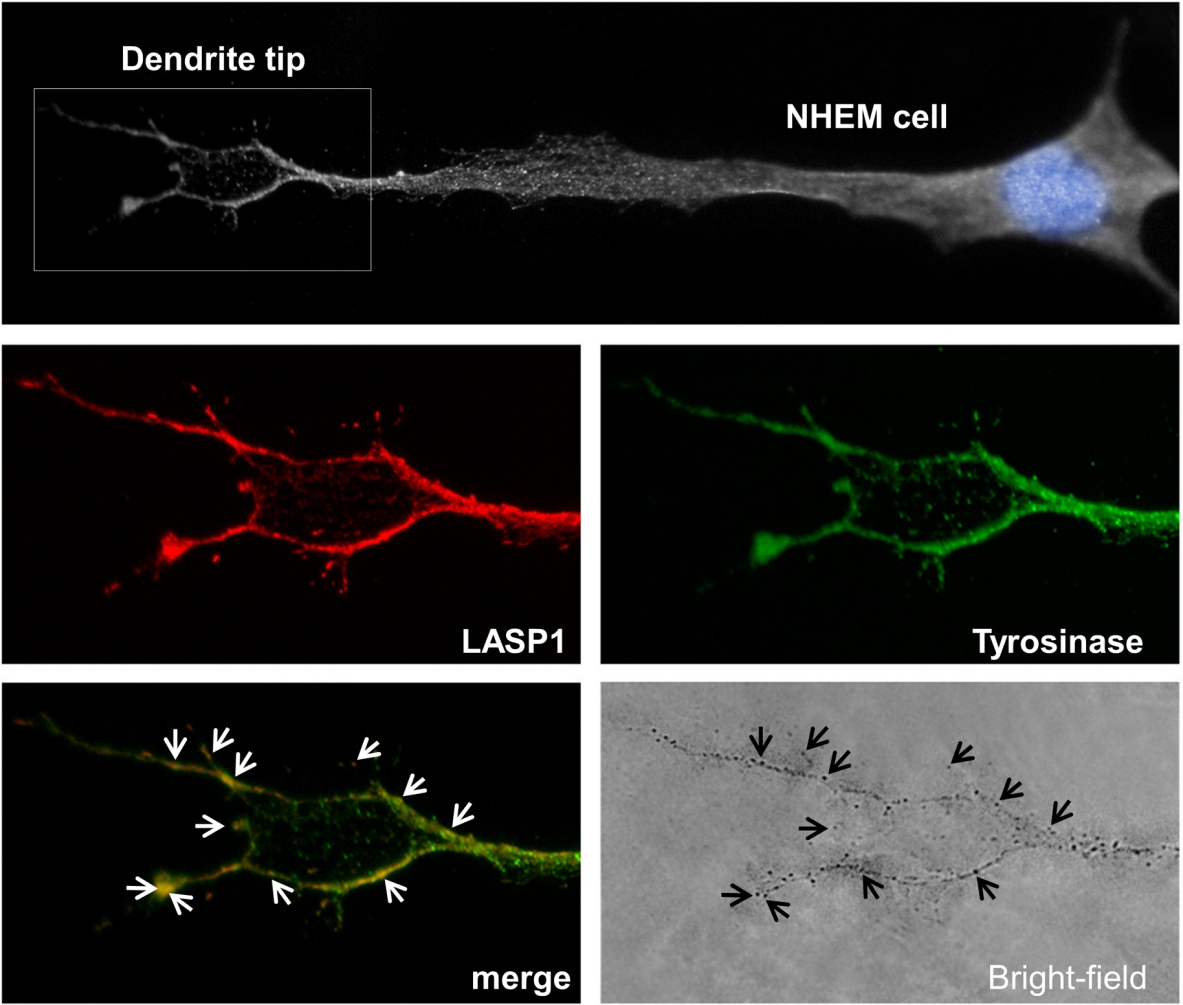
Immunofluorescence of LASP1 and tyrosinase in NHEM cells. Black and white image of a NHEM cell with highlighted blue nucleus and circled enlarged dendrite tip (upper panel). LASP1 (red) and tyrosinase (green) immunofluorescence at the dendrite tip of the NHEM cell (middle panels). Co-localization of the merged LASP1-tyrosinase complex (white arrows) with pointed melanosomes (black arrows) in the bright-field image at the dendrite tip of the NHEM cell (lower panels).

In line with these results, co-localization of the LASP1-dynamin complex with melanosomes at the tips of melanocyte dendrites is visible in Fig 6C. Furthermore, co-localization of LASP1 and tyrosinase (Fig 7) perfectly matches with the peripheral melanosomes, visible as dark spots in the bright-field illumination.

### LASP1 expression decreases in melanocytic cells during tumor progression

Cutaneous melanoma is a highly malignant neoplasm originating from melanocytes of the skin and bears a high risk for metastatic spread. As LASP1 is overexpressed in several cancer entities [14] it was tempting to speculate that the protein might also be involved in the development and progression of melanoma.

**Fig 8:**
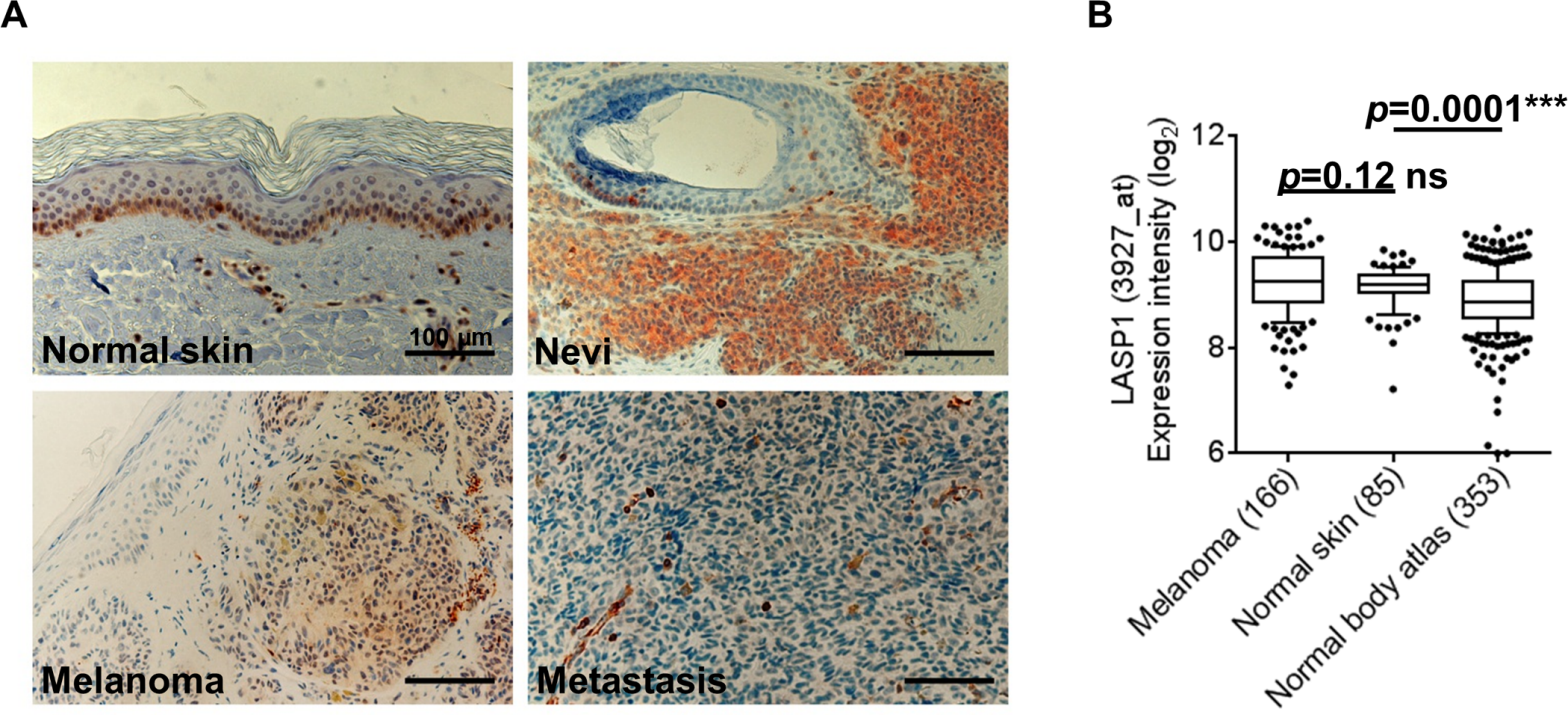
LASP1 expression in normal skin, melanocytic nevi, melanoma and metastases. (A) Representative immunohistochemical stainings of normal skin, melanocytic nevus, melanoma, and metastasis tissue samples for LASP1. Hematoxylin counterstaining; magnification 20x; Scale bars = 100 μm). (B) Gene expression patterns of LASP1 in skin tumor tissues: normal body atlas GSE3526; melanoma GSE10282, GSE15605, and GSE35640; normal skin GSE14905 and GSE13355. The number of samples is given in parentheses. *p* values < 0.05 were considered statistically significant (two-tailed student’s t-test with Welch’s correction).

We therefore assessed the LASP1 protein expression pattern by immunohistochemistry in specimens from 29 melanocytic nevi, 58 malignant melanoma samples and 20 melanoma metastases. Representative samples for the observed LASP1 immunoreactivity are shown in Fig 8A. For semi-quantitative assessment, the percentage of LASP1-positive cells and staining intensity were combined in the Immune Reactive Score (IRS), ranging from 0–12 [4, 18]. For normal skin the IRS turned out to be 1 with LASP1-positivity in the basal epidermal cell layer (Figs 1A, 1B and 8A) This is reflected by a moderate but significant (p = 0.0001) higher LASP1 mRNA level in normal skin as compared to a normal body atlas retrieved from the GEO (Fig 8B).

For statistical evaluation of tumor samples a LASP1-IRS ≥3 was classified as LASP1-positive. Based on this definition 62.0% of the benign nevi, 13.8% of the primary melanoma and 4.7% of the melanoma metastases were scored LASP1-positive (Table 1). Notably, these results, demonstrating loss of LASP1 expression in the course of melanoma progression, are all based on the presence of LASP1 in the cytoplasm since nuclear LASP1 staining, which is characteristic for many aggressive LASP1-positive tumors (breast, prostate and liver carcinoma, medulloblastoma [4, 32, 41, 42]) was not observed in any of the melanocytic tumor samples (Table 1).

**Table 1:** LASP1 expression in samples of melanocytic nevi, primary melanomas and melanoma metastases.

	LASP1 positive cells (IRS≥3)	LASP1 positive nuclei (≥10%)
**Nevi** (n = 29)	18 (62.0%)	0 (100%)
**Melanoma** (n = 58)	8 (13.8%)	0 (100%)
**Metastasis** (n = 20)	1 (4.7%)	0 (100%)

The observed low levels of LASP1 expression in melanoma and metastases are confirmed by microarray data set analysis, revealing no increase in LASP1 mRNA expression in melanoma when compared to normal skin (Fig 8B).

To further examine a possible role of LASP1 in melanoma, we correlated LASP1 expression levels with clinicopathological parameters of the patients (Table 2). There was no obvious correlation between LASP1 expression and age, gender, tumor depth (AJCC guidelines) or metastatic spread. For cancer-related death (CRD), a *p*-value of 0.05 is calculated. 75% of the LASP1-positive patients died (6 out of 8) compared to 38% of LASP1- negative patients (19 out of 50), assuming a trend to negative outcome for patients with LASP1 expression in melanoma. However, the overall number of deceased LASP1-positive individuals (n = 8) is too low for a statistically robust conclusion on CRD.

**Table 2:** Correlation of LASP1 expression to clinicopathological parameters in melanoma.

Parameter	Melanoma	LASP1 positive	LASP1 negative	*P* value
**Number of patients**	58	8	50	
**Sex**				0.81
Male	34	3	29	
Female	24	5	21	
**Age at primary diagnosis**				0.13
≤60	22	1	21	
>60	36	7	29	
Lymph node metastases *	30	6	24	0.18
Distant metastases *	28	6	22	0.11
Tumor depth * (AJCC)				0.10
1–2 mm (T2)	26	3	23	
2.1–4 mm (T3)	13	4	9	
>4 mm (T4)	19	1	18	
Cancer-related death *	25	6	19	0.05

* Tumor depth after AJCC guidelines.

** within 5 years of follow-up. Values of *p* were calculated by Chi-Square test; statistical significance is assumed when p<0.05.

### LASP1 is not localized in the nucleus in melanoma cell lines

**Fig 9:**
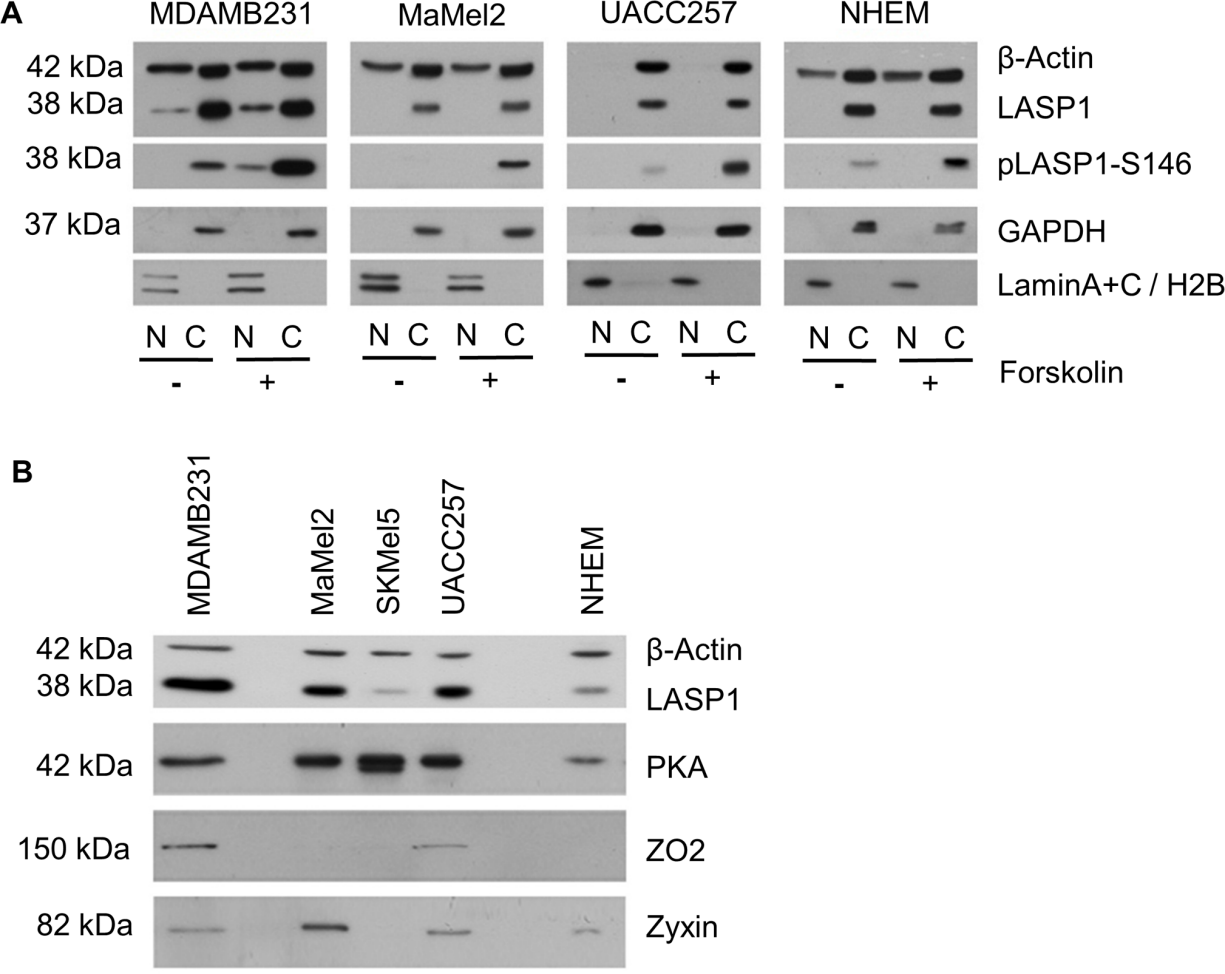
Nuclear and cytosolic LASP1 distribution. (A) Western blot analysis of LASP1 and pLASP1-S146 in cytosolic (C) and nuclear (N) fractions of MaMel2, UACC257, normal human epidermal melanocytes (NHEM) and breast cancer cell line MDAMB231 before and after forskolin stimulation. Phosphorylation of LASP1 by PKA does not influence cytosolic LASP1 localization in melanoma cell lines and NHEM. Purity and loading of the fractions were controlled by Western blot for β-actin, the cytosolic marker GAPDH and the nuclear markers Lamin A/C and histone H2B. (B) Western blot analysis of LASP1, ZO2 and PKA expression in MDA-MB 231 breast cancer cells compared to melanoma cell lines and NHEM. Loading was adjusted to similar β-actin levels.

A predominant nuclear localization of LASP1 is observed in several cancer entities [32, 43] and was reported to correlate with worse long-time survival in breast cancer [44]. To confirm the cytoplasmic LASP1 localization observed by immunohistochemistry of melanocytic tumors (Fig 1), WB analysis of cytosolic and nuclear fractions from MaMel2, UACC257, and NHEM in comparison to MDAMB231 breast cancer cells was performed. Indeed, LASP1 and pLASP1-S146 are exclusively detected in the cytosolic fraction of the melanoma cell lines and NHEMs, while in breast cancer cells a distinct nuclear LASP1 signal is seen (Fig 9A).

LASP1 is known to shuttle between the cytosol and the nucleus in a phosphorylation-dependent manner that requires binding of the protein to ZO2 [10]. We therefore analyzed i) the expression of potentially involved shuttle proteins in melanoma cells and ii) potential nuclear translocation of LASP1 after forskolin-triggered protein kinase A (PKA) activation. As seen in Fig 9A, phosphorylation of LASP1 at Ser-146 by PKA is not inducing any translocation of the protein to the nucleus of the melanocytic cells as it is observed for the breast cancer cell line MDAMB231, which served as control. The nuclear absence of LASP1 in melanocytic cells may be explained by a lack of expression of the essential shuttle partner ZO2 in MaMel2, SKMel5 and NHEM cells, however, in UACC257 LASP1 stays in the cytoplasm although ZO2 is present (Fig 9B).

## Discussion

LASP1 was identified as a novel protein expressed by melanocytes and keratinocytes of the basal epidermal layer in healthy skin. Moreover, LASP1 is expressed in melanocytic nevi while in primary melanoma and in metastases LASP1 levels are reduced. Keratinocytes in suprabasal layers of the epidermis are LASP1-negative (Figs 1 and 8).

The LASP1-positive epidermal basal cells demonstrate nuclear LASP1 accumulation (Fig 1B). In earlier studies, positive nuclear LASP1 staining was associated with aggressive, proliferative cell growth and a transcriptional function or a role in cell cycle control for LASP1 in the nucleus is discussed [32, 44]. In view of these data, the nuclear localization of LASP1 in this highly proliferative cell layer is explainable.

Up to this study, LASP1 has been depicted as a protein being significantly upregulated in numerous tumor entities: colon [43], bladder [45], prostate [41] breast [4], liver [42], gastric [46], renal [47], oral malignant tumors [48] as well as medulloblastoma [32]. Several of these studies demonstrate that LASP1 expression and nuclear localization are positively correlated with malignancy, tumor grade and metastatic lymph node status [32, 41, 44]. Melanoma is the first tumor tested so far, that is showing no LASP1 overexpression and obviously no involvement in the development and progression of melanoma.

The primary function of melanocytes is the synthesis of melanin pigments in specific organelles, termed melanosomes. In a first step, vesicles containing melanosomal proteins are bud from the ER. The vesicles are moved forward to the Golgi apparatus around the nucleus. Then, mature melanosomes are transported along microtubules and actin filaments towards the surface of the melanocyte. During this transport the melanosomes pass through four stages: Non-pigmented stage I vacuolar early endosomes; melanosomes stage II with fibrillar striations; pigmented melanosomes at stage III and IV with predominant expression of tyrosinase and TRP1. Melanosomes at stage IV are released into the extracellular space and phagocytosed by keratinocytes [1, 2, 39, 49].

In 2006, a proteomic characterization of melanosomes by tandem mass spectrometry listed LASP1 and dynamin as putative proteins in these organelles, however, the presence of the proteins was not validated [37]. Our sucrose gradient data now revealed co-localization of LASP1 with dynamin, tyrosinase and TRP1 in melanosomes at stage IV. Although dynamin plays a pivotal role in vesicle trafficking from endosomes to the Golgi compartment, our immunofluorescence data clearly demonstrate the presence of the verified LASP1-dynamin complex only along the melanocyte dendrite tips, assuming a role of this complex in melanosome vesicle release.

**Fig 10:**
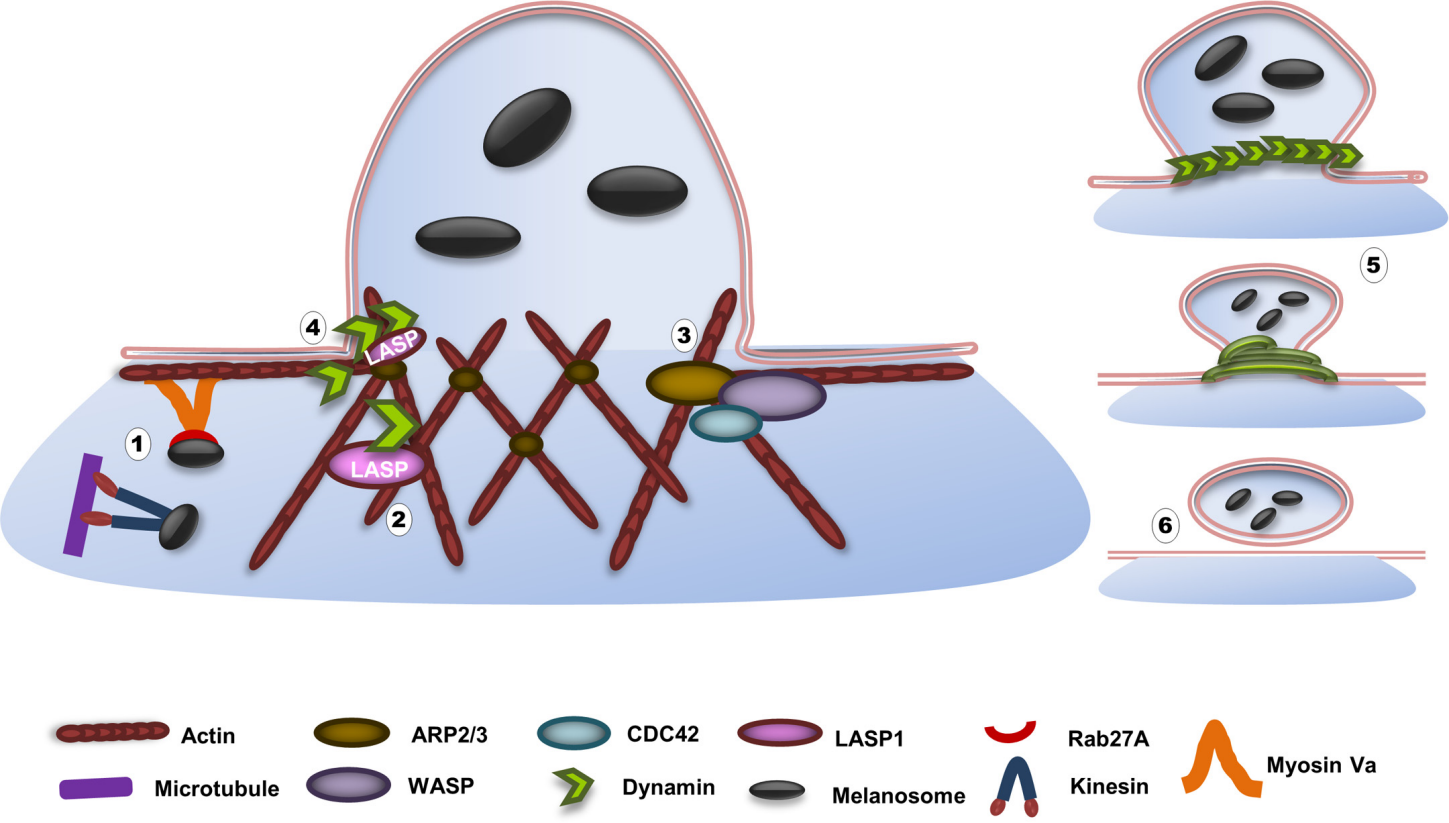
Proposed model for LASP1 involvement in actin-dynamin- mediated melanosome vesicle scission at melanocyte dendrite tips. In anterograde melanosomal transport mature melanosomes move along microtubules by means of the motor protein kinesin and are transferred towards the cell periphery. Once at the periphery, track switching from microtubules to actin filaments occurs, a process mediated by Rab27A and molecular motor protein myosin Va (1). In this fashion microtubule, actin filaments and motor systems co-operate to promote melanosome transport and retention of the organelle in peripheral dendrites. LASP1 and dynamin are present at the actin mesh at the plasma membrane. LASP1 binds to actin and dynamin through its nebulin repeat and SH3 domain, respectively. Dynamin exists as a dimer and binds to actin through the stalk region (2). WASP- and CDC42-mediated activation of ARP2/3 lead to the branched polymerization of actin filaments towards the release site. Actin, together with other motor proteins, pushes the plasma membrane and enhances membrane invagination (3). As an adaptor/scaffolding protein, LASP1 recruits and positions dynamin at the tubular membrane (4). Subsequent polymerization of dynamin around the membrane in a helical manner and GTP hydrolysis results in membrane constriction and melanosome vesicle scission at dendrite tips (5 and 6).

According to the latest data on melanosome transfer [1], melanosomes are packed in vesicles, budded off from melanocyte dendrites, released into the extracellular space, and then are phagocytosed by keratinocytes [2]. The mechanisms of vesicle formation and scission, either into the plasma membrane (endocytosis) or out of the Golgi apparatus, (secretion) are well characterized [50] and the involvement of F-actin and dynamin in membrane fission has been shown in many studies [51]. In view of these models, we are hypothesizing a possible participation of LASP1 in F-actin-dynamin mediated vesicle budding in melanocytes (Fig 10).

During exocytosis, dynamin forms helical polymers around the membrane neck of nascent exocytic buds at the plasma membrane and by constriction of the helix, the membrane neck will break [52] resulting in shedding of melanosomes-containing vesicles into the extracellular matrix [53]. This process involves several scaffolding proteins that connect the exocytotic machinery to the actin cytoskeleton of the cell, among them WASP, Arp2/3, CDC42, dynamin and F-actin [50], the latter two known as LASP1 binding partners [35, 54]. Binding occurs between the N-terminal proline-rich repeat of dynamin with the SH3 domain of LASP1 [55] as well as between F-actin and the actin-binding nebulin repeats in LASP1 [56]. Disruption of this bridging complex by LASP1 knockdown is not crucial but leads to distinct reduction in melanosome vesicle budding, concomitant with increased cellular melanin levels, as observed in this study.

A comparable mechanism is discussed for the secretory HCl response in gastric parietal cells. Herein, protein kinase A (PKA) phosphorylation of LASP1 regulates the trafficking/activation of the H+/K+-ATPase vesicles. In LASP1 knockout mice, histamine-stimulated PKA activation induced a more robust acid secretory response [57]. The authors suggested a phosphorylation-dependent alteration of LASP1 binding to F-actin and discussed ezrin as well as dynamin as likely mediators, linking the vesicular trafficking machinery to the cytoskeleton. However, none of these postulated interactions was verified at that time.

In summary, we identified LASP1 as a hitherto unknown protein in melanocytes and as novel partner of dynamin in the complex process of melanosome vesicle release.

## References

[1] Wu X, Hammer JA. Melanosome transfer: it is best to give and receive. Curr Opin Cell Biol. 2014;29:1–7. Epub 2014/03/26. 10.1016/j.ceb.2014.02.003 24662021 PMC4130791

[2] Ando H, Niki Y, Ito M, Akiyama K, Matsui MS, Yarosh DB, et al Melanosomes are transferred from melanocytes to keratinocytes through the processes of packaging, release, uptake, and dispersion. J Invest Dermatol. 2012;132(4):1222–9. Epub 2011/12/23. 10.1038/jid.2011.413 . 22189785

[3] Akhmanova A, Hammer JA 3rd. Linking molecular motors to membrane cargo. Curr Opin Cell Biol. 2010;22(4):479–87. Epub 2010/05/15. 10.1016/j.ceb.2010.04.008 20466533 PMC3393125

[4] Grunewald TG, Kammerer U, Kapp M, Eck M, Dietl J, Butt E, et al Nuclear localization and cytosolic overexpression of LASP-1 correlates with tumor size and nodal-positivity of human breast carcinoma. BMC Cancer. 2007;7:198 Epub 2007/10/25. 10.1186/1471-2407-7-198 17956604 PMC2151952

[5] Grunewald TG, Butt E. The LIM and SH3 domain protein family: structural proteins or signal transducers or both? Mol Cancer. 2008;7:31 10.1186/1476-4598-7-31 18419822 PMC2359764

[6] Schreiber V, Masson R, Linares JL, Mattei MG, Tomasetto C, Rio MC. Chromosomal assignment and expression pattern of the murine Lasp-1 gene. Gene. 1998;207(2):171–5. Epub 1998/03/25. . 9511759 10.1016/s0378-1119(97)00622-7

[7] Butt E, Gambaryan S, Gottfert N, Galler A, Marcus K, Meyer HE. Actin binding of human LIM and SH3 protein is regulated by cGMP- and cAMP-dependent protein kinase phosphorylation on serine 146. J Biol Chem. 2003;278(18):15601–7. Epub 2003/02/07. 10.1074/jbc.M209009200 . 12571245

[8] Gray CH, McGarry LC, Spence HJ, Riboldi-Tunnicliffe A, Ozanne BW. Novel beta-propeller of the BTB-Kelch protein Krp1 provides a binding site for Lasp-1 that is necessary for pseudopodial extension. J Biol Chem. 2009;284(44):30498–507. Epub 2009/09/04. 10.1074/jbc.M109.023259 19726686 PMC2781604

[9] Li B, Zhuang L, Trueb B. Zyxin interacts with the SH3 domains of the cytoskeletal proteins LIM-nebulette and Lasp-1. J Biol Chem. 2004;279(19):20401–10. Epub 2004/03/09. 10.1074/jbc.M310304200 . 15004028

[10] Mihlan S, Reiss C, Thalheimer P, Herterich S, Gaetzner S, Kremerskothen J, et al Nuclear import of LASP-1 is regulated by phosphorylation and dynamic protein-protein interactions. Oncogene. 2013;32(16):2107–13. Epub 2012/06/06. 10.1038/onc.2012.216 . 22665060

[11] Frietsch JJ, Kastner C, Grunewald TG, Schweigel H, Nollau P, Ziermann J, et al LASP1 is a novel BCR-ABL substrate and a phosphorylation-dependent binding partner of CRKL in chronic myeloid leukemia. Oncotarget. 2014;5(14):5257–71. Epub 2014/06/11. 24913448 10.18632/oncotarget.2072 PMC4170624

[12] Raman D, Sai J, Neel NF, Chew CS, Richmond A. LIM and SH3 protein-1 modulates CXCR2-mediated cell migration. PLoS One. 2010;5(4):e10050 10.1371/journal.pone.0010050 20419088 PMC2856662

[13] Lin YH, Park ZY, Lin D, Brahmbhatt AA, Rio MC, Yates JR 3rd, et al Regulation of cell migration and survival by focal adhesion targeting of Lasp-1. J Cell Biol. 2004;165(3):421–32. 10.1083/jcb.200311045 15138294 PMC2172195

[14] Orth MF, Cazes A, Butt E, Grunewald TG. An update on the LIM and SH3 domain protein 1 (LASP1): a versatile structural, signaling, and biomarker protein. Oncotarget. 2015;6(1):26–42. Epub 2015/01/27. . 25622104 10.18632/oncotarget.3083 PMC4381576

[15] Okamoto CT, Li R, Zhang Z, Jeng YY, Chew CS. Regulation of protein and vesicle trafficking at the apical membrane of epithelial cells. J Control Release. 2002;78(1–3):35–41. Epub 2002/01/05. . 11772447 10.1016/s0168-3659(01)00479-5

[16] Chew CS, Parente JA Jr., Chen X, Chaponnier C, Cameron RS. The LIM and SH3 domain-containing protein, lasp-1, may link the cAMP signaling pathway with dynamic membrane restructuring activities in ion transporting epithelia. J Cell Sci. 2000;113 (Pt 11):2035–45. Epub 2000/05/12. . 10806114 10.1242/jcs.113.11.2035

[17] Houben R, Adam C, Baeurle A, Hesbacher S, Grimm J, Angermeyer S, et al An intact retinoblastoma protein-binding site in Merkel cell polyomavirus large T antigen is required for promoting growth of Merkel cell carcinoma cells. Int J Cancer. 2012;130(4):847–56. Epub 2011/03/18. 10.1002/ijc.26076 . 21413015

[18] Remmele W, Stegner HE. [Recommendation for uniform definition of an immunoreactive score (IRS) for immunohistochemical estrogen receptor detection (ER-ICA) in breast cancer tissue]. Der Pathologe. 1987;8(3):138–40. . 3303008

[19] Marshall ES, Matthews JH, Shaw JH, Nixon J, Tumewu P, Finlay GJ, et al Radiosensitivity of new and established human melanoma cell lines: comparison of [3H]thymidine incorporation and soft agar clonogenic assays. Eur J Cancer. 1994;30A(9):1370–6. Epub 1994/01/01. . 7999427 10.1016/0959-8049(94)90188-0

[20] Gobeil S, Zhu X, Doillon CJ, Green MR. A genome-wide shRNA screen identifies GAS1 as a novel melanoma metastasis suppressor gene. Genes Dev. 2008;22(21):2932–40. Epub 2008/11/05. 10.1101/gad.1714608 18981472 PMC2577790

[21] Ugurel S, Thirumaran RK, Bloethner S, Gast A, Sucker A, Mueller-Berghaus J, et al B-RAF and N-RAS mutations are preserved during short time in vitro propagation and differentially impact prognosis. PLoS One. 2007;2(2):e236 Epub 2007/02/22. 10.1371/journal.pone.0000236 17311103 PMC1794595

[22] Houben R, Hesbacher S, Schmid CP, Kauczok CS, Flohr U, Haferkamp S, et al High-level expression of wild-type p53 in melanoma cells is frequently associated with inactivity in p53 reporter gene assays. PLoS One. 2011;6(7):e22096 Epub 2011/07/16. 10.1371/journal.pone.0022096 21760960 PMC3132323

[23] Roth RB, Hevezi P, Lee J, Willhite D, Lechner SM, Foster AC, et al Gene expression analyses reveal molecular relationships among 20 regions of the human CNS. Neurogenetics. 2006;7(2):67–80. 10.1007/s10048-006-0032-6 . 16572319

[24] Irizarry RA, Hobbs B, Collin F, Beazer-Barclay YD, Antonellis KJ, Scherf U, et al Exploration, normalization, and summaries of high density oligonucleotide array probe level data. Biostatistics. 2003;4(2):249–64. 10.1093/biostatistics/4.2.249 . 12925520

[25] Dai M, Wang P, Boyd AD, Kostov G, Athey B, Jones EG, et al Evolving gene/transcript definitions significantly alter the interpretation of GeneChip data. Nucleic Acids Res. 2005;33(20):e175 10.1093/nar/gni179 16284200 PMC1283542

[26] Grunewald TG, Willier S, Janik D, Unland R, Reiss C, Prazeres da Costa O, et al The Zyxin-related protein thyroid receptor interacting protein 6 (TRIP6) is overexpressed in Ewing's sarcoma and promotes migration, invasion and cell growth. Biol Cell. 2013;105(11):535–47. Epub 2013/09/17. 10.1111/boc.201300041 . 24033704

[27] Willier S, Butt E, Grunewald TG. Lysophosphatidic acid (LPA) signalling in cell migration and cancer invasion: a focussed review and analysis of LPA receptor gene expression on the basis of more than 1700 cancer microarrays. Biol Cell. 2013;105(8):317–33. 10.1111/boc.201300011 . 23611148

[28] Watabe H, Kushimoto T, Valencia JC, Hearing VJ. Isolation of melanosomes. Curr Protoc Cell Biol. 2005;Chapter 3:Unit 3 14. Epub 2008/01/30. 10.1002/0471143030.cb0314s26 . 18228474

[29] Grunewald TG, Kammerer U, Winkler C, Schindler D, Sickmann A, Honig A, et al Overexpression of LASP-1 mediates migration and proliferation of human ovarian cancer cells and influences zyxin localisation. Br J Cancer. 2007;96(2):296–305. Epub 2007/01/11. 10.1038/sj.bjc.6603545 17211471 PMC2359999

[30] Stolting M, Wiesner C, van Vliet V, Butt E, Pavenstadt H, Linder S, et al Lasp-1 regulates podosome function. PLoS One. 2012;7(4):e35340 Epub 2012/04/20. 10.1371/journal.pone.0035340 22514729 PMC3325968

[31] Wang B, Feng P, Xiao Z, Ren EC. LIM and SH3 protein 1 (Lasp1) is a novel p53 transcriptional target involved in hepatocellular carcinoma. J Hepatol. 2009;50(3):528–37. Epub 2009/01/22. 10.1016/j.jhep.2008.10.025 . 19155088

[32] Traenka C, Remke M, Korshunov A, Bender S, Hielscher T, Northcott PA, et al Role of LIM and SH3 protein 1 (LASP1) in the metastatic dissemination of medulloblastoma. Cancer Res. 2010;70(20):8003–14. Epub 2010/10/07. 10.1158/0008-5472.CAN-10-0592 . 20924110

[33] Tang R, Kong F, Hu L, You H, Zhang P, Du W, et al Role of hepatitis B virus X protein in regulating LIM and SH3 protein 1 (LASP-1) expression to mediate proliferation and migration of hepatoma cells. Virol J. 2012;9:163 Epub 2012/08/18. 10.1186/1743-422X-9-163 22897902 PMC3459728

[34] Zhao T, Ren H, Li J, Chen J, Zhang H, Xin W, et al. LASP1 is a HIF-1alpha target gene critical for metastasis of pancreatic cancer. Cancer Res. 2014. Epub 2014/11/12. 10.1158/0008-5472.CAN-14-2040 . 25385028 PMC4286473

[35] Chew CS, Chen X, Parente JA Jr., Tarrer S, Okamoto C, Qin HY. Lasp-1 binds to non-muscle F-actin in vitro and is localized within multiple sites of dynamic actin assembly in vivo. J Cell Sci. 2002;115(Pt 24):4787–99. Epub 2002/11/15. . 12432067 10.1242/jcs.00174

[36] Watabe H, Valencia JC, Le Pape E, Yamaguchi Y, Nakamura M, Rouzaud F, et al Involvement of dynein and spectrin with early melanosome transport and melanosomal protein trafficking. J Invest Dermatol. 2008;128(1):162–74. Epub 2007/08/10. 10.1038/sj.jid.5701019 17687388 PMC2167631

[37] Chi A, Valencia JC, Hu ZZ, Watabe H, Yamaguchi H, Mangini NJ, et al Proteomic and bioinformatic characterization of the biogenesis and function of melanosomes. J Proteome Res. 2006;5(11):3135–44. Epub 2006/11/04. 10.1021/pr060363j . 17081065

[38] Schmid SL. Clathrin-coated vesicle formation and protein sorting: an integrated process. Annu Rev Biochem. 1997;66:511–48. Epub 1997/01/01. 10.1146/annurev.biochem.66.1.511 . 9242916

[39] Raposo G, Marks MS. Melanosomes—dark organelles enlighten endosomal membrane transport. Nat Rev Mol Cell Biol. 2007;8(10):786–97. Epub 2007/09/20. 10.1038/nrm2258 17878918 PMC2786984

[40] Kushimoto T, Basrur V, Valencia J, Matsunaga J, Vieira WD, Ferrans VJ, et al A model for melanosome biogenesis based on the purification and analysis of early melanosomes. Proc Natl Acad Sci U S A. 2001;98(19):10698–703. Epub 2001/08/30. 10.1073/pnas.191184798 11526213 PMC58529

[41] Hailer A, Grunewald TG, Orth M, Reiss C, Kneitz B, Spahn M, et al Loss of tumor suppressor mir-203 mediates overexpression of LIM and SH3 Protein 1 (LASP1) in high-risk prostate cancer thereby increasing cell proliferation and migration. Oncotarget. 2014;5(12):4144–53. Epub 2014/07/02. 24980827 10.18632/oncotarget.1928 PMC4147312

[42] Wang H, Li W, Jin X, Cui S, Zhao L. LIM and SH3 protein 1, a promoter of cell proliferation and migration, is a novel independent prognostic indicator in hepatocellular carcinoma. Eur J Cancer. 2013;49(4):974–83. Epub 2012/10/23. 10.1016/j.ejca.2012.09.032 . 23084841

[43] Zhao L, Wang H, Liu C, Liu Y, Wang X, Wang S, et al Promotion of colorectal cancer growth and metastasis by the LIM and SH3 domain protein 1. Gut. 2010;59(9):1226–35. 10.1136/gut.2009.202739 . 20660701

[44] Frietsch JJ, Grunewald TG, Jasper S, Kammerer U, Herterich S, Kapp M, et al Nuclear localisation of LASP-1 correlates with poor long-term survival in female breast cancer. Br J Cancer. 2010;102(11):1645–53. 10.1038/sj.bjc.6605685 20461080 PMC2883150

[45] Ardelt P, Grunemay N, Strehl A, Jilg C, Miernik A, Kneitz B, et al LASP-1, a novel urinary marker for detection of bladder cancer. Urol Oncol. 2013;31(8):1591–8. Epub 2012/04/07. 10.1016/j.urolonc.2012.02.002 . 22481019

[46] Zheng J, Yu S, Qiao Y, Zhang H, Liang S, Wang H, et al LASP-1 promotes tumor proliferation and metastasis and is an independent unfavorable prognostic factor in gastric cancer. J Cancer Res Clin Oncol. 2014;140(11):1891–9. Epub 2014/07/06. 10.1007/s00432-014-1759-3 . 24990592 PMC11823780

[47] Yang F, Zhou X, Du S, Zhao Y, Ren W, Deng Q, et al LIM and SH3 domain protein 1 (LASP-1) overexpression was associated with aggressive phenotype and poor prognosis in clear cell renal cell cancer. PLoS One. 2014;9(6):e100557 Epub 2014/06/24. 10.1371/journal.pone.0100557 24955835 PMC4067378

[48] Shimizu F, Shiiba M, Ogawara K, Kimura R, Minakawa Y, Baba T, et al Overexpression of LIM and SH3 Protein 1 leading to accelerated G2/M phase transition contributes to enhanced tumourigenesis in oral cancer. PLoS One. 2013;8(12):e83187 Epub 2014/01/05. 10.1371/journal.pone.0083187 24386158 PMC3873298

[49] Wasmeier C, Hume AN, Bolasco G, Seabra MC. Melanosomes at a glance. J Cell Sci. 2008;121(Pt 24):3995–9. Epub 2008/12/06. 10.1242/jcs.040667 . 19056669

[50] Anitei M, Hoflack B. Bridging membrane and cytoskeleton dynamics in the secretory and endocytic pathways. Nat Cell Biol. 2012;14(1):11–9. Epub 2011/12/24. 10.1038/ncb2409 . 22193159

[51] Faelber K, Held M, Gao S, Posor Y, Haucke V, Noe F, et al Structural insights into dynamin-mediated membrane fission. Structure. 2012;20(10):1621–8. Epub 2012/10/16. 10.1016/j.str.2012.08.028 . 23063009

[52] Morlot S, Roux A. Mechanics of dynamin-mediated membrane fission. Annu Rev Biophys. 2013;42:629–49. Epub 2013/04/02. 10.1146/annurev-biophys-050511-102247 . 23541160 PMC4289195

[53] Ando H, Niki Y, Yoshida M, Ito M, Akiyama K, Kim JH, et al Involvement of pigment globules containing multiple melanosomes in the transfer of melanosomes from melanocytes to keratinocytes. Cell Logist. 2011;1(1):12–20. Epub 2011/06/21. 10.4161/cl.1.1.13638 21686100 PMC3109459

[54] Schreiber V, Moog-Lutz C, Regnier CH, Chenard MP, Boeuf H, Vonesch JL, et al Lasp-1, a novel type of actin-binding protein accumulating in cell membrane extensions. Mol Med. 1998;4(10):675–87. Epub 1998/12/16. 9848085 PMC2230251

[55] Keicher C, Gambaryan S, Schulze E, Marcus K, Meyer HE, Butt E. Phosphorylation of mouse LASP-1 on threonine 156 by cAMP- and cGMP-dependent protein kinase. Biochem Biophys Res Commun. 2004;324(1):308–16. Epub 2004/10/07. 10.1016/j.bbrc.2004.08.235 . 15465019

[56] Panaviene Z, Moncman CL. Linker region of nebulin family members plays an important role in targeting these molecules to cellular structures. Cell Tissue Res. 2007;327(2):353–69. Epub 2006/12/21. 10.1007/s00441-006-0305-2 . 17177073

[57] Chew CS, Chen X, Bollag RJ, Isales C, Ding KH, Zhang H. Targeted disruption of the Lasp-1 gene is linked to increases in histamine-stimulated gastric HCl secretion. Am J Physiol Gastrointest Liver Physiol. 2008;295(1):G37–G44. Epub 2008/05/17. 10.1152/ajpgi.90247.2008 18483181 PMC2494726

